# Emerging roles of spliceosome in cancer and immunity

**DOI:** 10.1007/s13238-021-00856-5

**Published:** 2021-07-01

**Authors:** Hui Yang, Bruce Beutler, Duanwu Zhang

**Affiliations:** 1 Department of Neurosurgery, Huashan Hospital, Shanghai Key laboratory of Brain Function Restoration and Neural Regeneration, MOE Frontiers Center for Brain Science, Institute for Translational Brain Research, Shanghai Medical College, Fudan University, Shanghai, 200032 China; 2 Center for the Genetics of Host Defense, University of Texas Southwestern Medical Center, Dallas, TX 75390 USA; 3 Children’s Hospital of Fudan University, and Shanghai Key Laboratory of Medical Epigenetics, International Co-laboratory of Medical Epigenetics and Metabolism, Ministry of Science and Technology, Institutes of Biomedical Sciences, Fudan University, Shanghai, 200032 China

**Keywords:** spliceosome, splicing, cancer, innate immunity, immune dysregulation

## Abstract

Precursor messenger RNA (pre-mRNA) splicing is catalyzed by an intricate ribonucleoprotein complex called the spliceosome. Although the spliceosome is considered to be general cell “housekeeping” machinery, mutations in core components of the spliceosome frequently correlate with cell- or tissue-specific phenotypes and diseases. In this review, we expound the links between spliceosome mutations, aberrant splicing, and human cancers. Remarkably, spliceosome-targeted therapies (STTs) have become efficient anti-cancer strategies for cancer patients with splicing defects. We also highlight the links between spliceosome and immune signaling. Recent studies have shown that some spliceosome gene mutations can result in immune dysregulation and notable phenotypes due to mis-splicing of immune-related genes. Furthermore, several core spliceosome components harbor splicing-independent immune functions within the cell, expanding the functional repertoire of these diverse proteins.

## Introduction

Pre-mRNA is transcribed from a gene’s DNA template. The pre-mRNA undergoes splicing to remove introns, forming mature messenger RNA (mRNA) that directs the synthesis of the protein during translation. Intron sequences in the pre-mRNAs contain several conserved sequences that facilitate proper splicing, including a 5′ splice site, a 3′ splice site, and the branch point typically 18 to 40 base pairs upstream of the 3′ splice site. Higher eukaryotes also have a polypyrimidine tract (PPT) following the branch point that is essential for recruiting splicing factors to the 3′ splice site (Taylor and Lee 2019). During splicing, two transesterification reactions remove introns from pre-mRNA (Moore 1993). In the first reaction, the 5′ splice site is cleaved and the 5′ end of the intron ligates to the branch adenosine of the intron. In the second reaction, the 5′ and the 3′ exons are ligated after the 3′ splice site is cleaved by the 3′ OH group of the 5′ exon.

In addition to constitutive splicing, a single pre-mRNA can be alternatively spliced, often in a tissue- or cellular condition-specific manner. In alternative splicing, exons can be skipped or extended as well as introns retained to produce different forms of the mRNA. Except for isoforms that undergo nonsense-mediated mRNA decay or are retained in the nucleus, alternatively spliced mRNAs can be translated into multiple protein products that can have unique functions (Nilsen and Graveley 2010).

The splicing of pre-mRNA is executed by the spliceosome, a multi-megadalton ribonucleoprotein complex. Although all eukaryotic cells have spliceosomes, mutations in core components of the spliceosome frequently correlate with specific phenotypes and diseases. In this review, we discuss the important roles of spliceosome components and splicing factors in cancer and immunity.

## The eukaryote spliceosome

**Figure 1:**
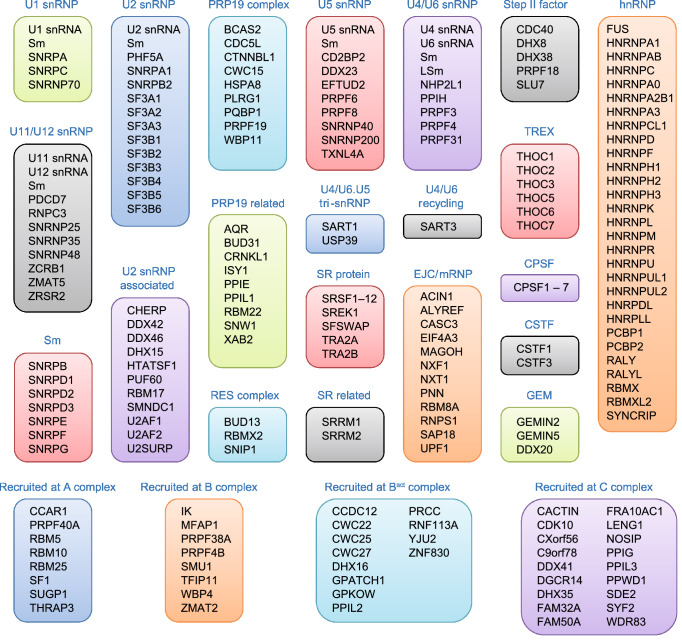
The human spliceosome machinery. Human spliceosome consists of several snRNPs. Each snRNP is composed of a uridine-rich small nuclear RNA (U snRNA), Sm proteins (i.e., SNRPB/B′, D1, D2, D3, E, F, and G) or LSm proteins (i.e., LSm2–8), and a variable number of particle-specific proteins. The U4/U6.U5 tri-snRNP contains two sets of Sm proteins and one set of LSm proteins. Classification is based on molecular features (e.g., SR proteins), association with stable spliceosome sub-complexes like the snRNPs or PRP19 complex, and other common designations (e.g., hnRNP, step II factors). Proteins that do not associate specifically with a given complex, such as general RNA binding factors, are not included in this figure. All protein names listed are official symbol from the National Center for Biotechnology Information (NCBI) database. The figure was illustrated based on the complex analysis results that were collected in the Spliceosome Database (Cvitkovic and Jurica 2013). Abbreviations: RES, retention and splicing; SR, serine and arginine-rich; EJC, exon junction complex; mRNP, messenger ribonucleoprotein; TREX, transcription-export; CPSF, cleavage and polyadenylation specificity factor; CSTF, cleavage stimulation factor; GEM, gemini of coiled bodies; hnRNP, heterogeneous nuclear ribonucleoprotein

The eukaryote spliceosome consists of several small nuclear ribonucleoproteins (snRNPs). Each snRNP is composed of a uridine-rich small nuclear RNA (U snRNA), Sm proteins (i.e., SNRPB/B′, D1, D2, D3, E, F, and G), and a variable number of associated proteins (Will 2006; Fabrizio et al. 2009; Cvitkovic and Jurica 2013) (Fig. 1). At any point during splicing, over 170 proteins associate with the spliceosome (Jurica and Moore 2003; Wahl et al. 2009; Cvitkovic and Jurica 2013). Approximately 45 of the proteins are components of the snRNPs, while the other proteins are non-snRNP proteins that mediate spliceosome assembly, pre-mRNA splice site recognition, and pre-mRNA binding (Matlin and Moore 2007; Staley and Woolford 2009). Several of the spliceosome-associated proteins have redundant functions and/or are loosely associated, indicating that each spliceosome-associated protein is not required to splice every pre-mRNA substrate (Wahl et al. 2009).

**Figure 2:**
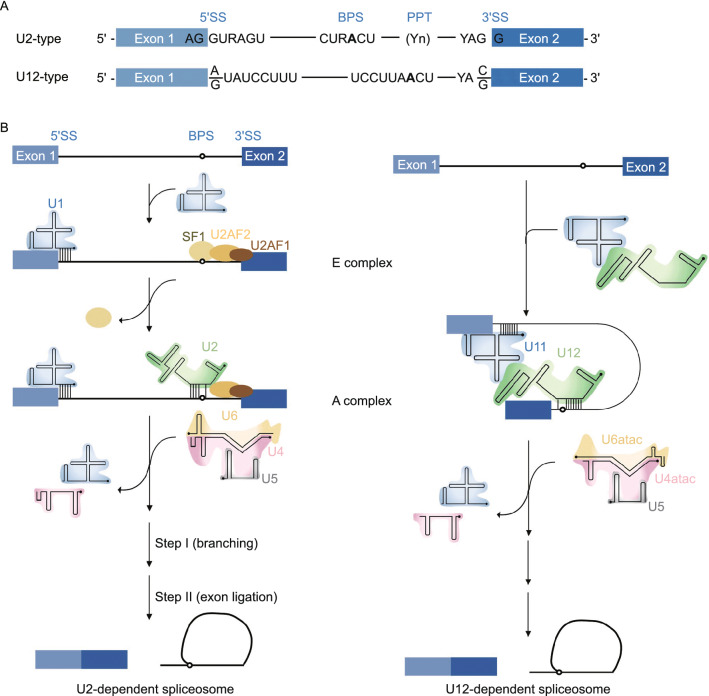
U2-dependent (major) spliceosome versus U12-dependent (minor) spliceosome. (A) The main determinants for distinguishing U2- and U12-type introns are the 5′ splice site (5′SS) and branch point sequence (BPS). U12-type introns are subdivided into AT-AC or GT-AG minor introns based on their terminal dinucleotides. (B) U2- and U12-type introns are recognized differently by their respective spliceosomes. U2-type introns are initially recognized by the U1 snRNP binding to the 5′SS, splicing factor 1 (SF1) binding to the BPS and U2 auxiliary factor (U2AF2/1) heterodimer binding to the polypyrimidine tract (PPT) and 3′SS, respectively. Subsequently, the U2 snRNA associates with the BPS and displaces SF1, converting the E complex to A complex. In contrast to the U2-type introns, the 5′SS and BPS of U12-type introns are recognized cooperatively by the U11 and U12 snRNAs of the di-snRNP, respectively, thereby forming the U12-type A complex. The following steps in the splicing process are similar between the U2- and U12-dependent pathway, and lead to similar catalytic structures and catalytic reactions of splicing

Eukaryotic cells have two types of spliceosomes: the U2-dependent (major) spliceosome and the U12-dependent (minor) spliceosome (Patel and Steitz 2003) (Fig. 2). Both spliceosomes are structurally and functionally similar in that each has five U snRNAs, but the types of snRNAs in each of the spliceosomes are unique. The U2 spliceosome contains the U1, U2, U4, U5, and U6 snRNAs, while the U12 spliceosome contains the unique U11, U12, U4atac, and U6atac snRNAs; both the U2 and U12 spliceosomes contain U5 snRNA (Patel and Steitz 2003; Turunen et al. 2013; Scotti and Swanson 2016; Shi 2017a, b). Most of the spliceosome-associated proteins are shared between the U2 and U12 spliceosomes, except those associated with the unique snRNPs (i.e., U1 and U2 in the U2 spliceosome and U11 and U12 in the U12 spliceosome) (Verma et al. 2018). The U2 spliceosome processes approximately 95.5% of all U2-type introns (Turunen et al. 2013), while the U12 spliceosome functions in the splicing of rare U12-type introns. U12-type introns occur in approximately 0.35% of all human introns in 700 to 800 genes. Most of these genes encode proteins that function in DNA replication and repair, translation, RNA processing, transcription, cytoskeletal organization, voltage-gated ion channel activity, and vesicular transport (Burge et al. 1998).

**Figure 3:**
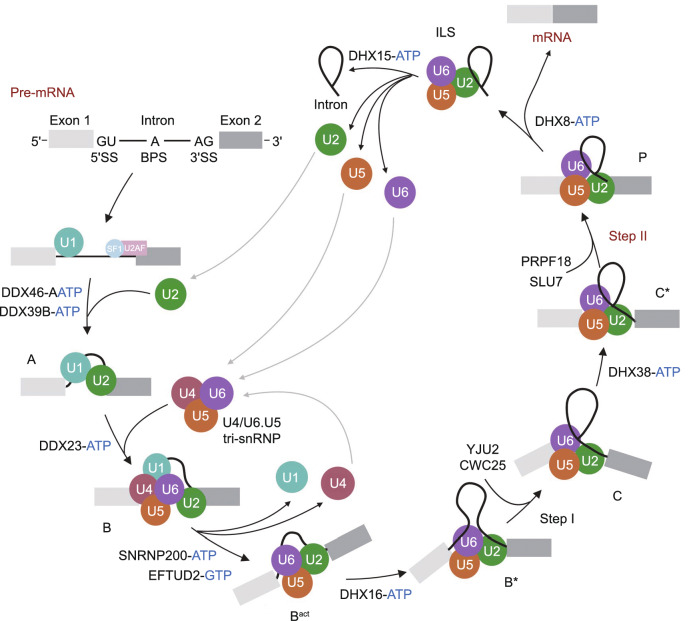
Eukaryotic U2-dependent splicing cycle. The 5′SS, BPS and 3′SS are first recognized by the U1 snRNP, SF1 and U2AF, respectively, forming an early spliceosome (E complex). SF1 is displaced by the U2 snRNP to form the pre-spliceosome (A complex), which associates with the U4/U6.U5 tri-snRNP to assemble into the pre-catalytic spliceosome (B complex). B complex undergoes a series of rearrangements to form a catalytically active B^act^ complex and then B* complex, which carries out the first catalytic step of splicing, generating C complex. C complex undergoes additional rearrangements and then carries out the second catalytic step, resulting in a post-catalytic spliceosome (P complex) that contains the lariat intron and spliced exons. Release of the spliced exons from P complex generates the intron lariat spliceosome (ILS complex). Finally, the U2, U5 and U6 snRNPs are released from the mRNP particle and recycled for additional rounds of splicing. Each complex has a unique composition, and conversions between complexes are driven by highly conserved RNA-dependent ATPase/helicases (including DDX46, DDX39B, DDX23, SNRNP200, DHX16, DHX38, DHX8 and DHX15, and the GTPase EFTUD2)

There are at least seven configurations of the spliceosome complex during splicing: the pre-catalytic complex (B), the activated complex (B^act^), the catalytically activated complex (B*), the catalytic step I spliceosome (C), the step II catalytically activated complex (C*), the post-catalytic complex (P), and the intron lariat spliceosome (ILS) (Fabrizio et al. 2009; Wahl et al. 2009) (Fig. 3). In the initial spliceosome complex (designated the E complex), the U1 snRNP (U11 snRNP in the U12 spliceosome) is recruited to the 5′ splice site allowing binding of the U1 (U11) snRNA (Taylor and Lee 2019). Non-snRNP factors such as splicing factor 1 (SF1) and U2 auxiliary factor (U2AF) interact with the branch point and the 3′ splice site, respectively. Subsequently, the U2 snRNP (U12 snRNP in the U12 spliceosome) associates with the branch point and displaces SF1 via binding of the U2 (U12) snRNA to the branch point, forming the prespliceosome (alternatively, the A complex) (Wassarman and Steitz 1992). A U4/U6.U5 tri-snRNP complex (the U4atac/U6atac.U5 tri-snRNP complex in the U12 spliceosome) is recruited to the prespliceosome to subsequently form the B complex. Rearrangements in RNA-RNA and RNA-protein interactions result in destabilization of the U1 (U11) and U4 (U4atac) snRNPs. The 5′ end of the U6 (U6atac) snRNA base pairs with the 5′ splice site, the U2 (U12) snRNA forms a duplex with the branch point, and the U1 (U11) and U4 (U4atac) snRNAs are displaced, resulting in formation of the B^act^ complex. The B^act^ complex is catalytically activated by the DEAH-box ATP-dependent RNA helicase DHX16 to form the B* complex, which catalyzes the first transesterification reaction during splicing. The first splicing reaction results in spliceosome rearrangement to form the C complex. Subsequently, the DEAH-box ATPase DHX38 catalytically activates the C complex to form the C* complex, which catalyzes the second transesterification reaction. After the reaction, the P complex contains ligated exons (mRNA) and the excised lariat-intron. The DEAH-box ATPase DHX8 releases the spliced mRNA from the P complex, which forms the ILS. The ILS dissociates with assistance from the DEAH-box ATPase DHX15, and the snRNPs are able to be reused in additional splicing (Will and Luhrmann, 2011) (Fig. 3).

## Links between spliceosome mutations and diseases

Proper recognition of intron sequences by the spliceosome is essential for intron removal. Mutations in core consensus sequences (i.e., 5′ or 3′ splice sites or the branch point sequence), exon and intron splicing enhancer or silencer elements, splicing factors, spliceosome assembly factors, and spliceosome components can result in pre-mRNA processing defects, truncated or aberrant protein products, and/or increased nonsense-mediated decay of the affected mRNAs (Wang and Cooper 2007; Turunen et al. 2008; Singh and Cooper 2012; Inoue et al. 2016; Ruzickova and Stanek 2017; Verma et al. 2018).

Aberrant pre-mRNA splicing due to mutations in snRNP components and snRNP-associated proteins are linked to several human diseases (Table 1) (Novoyatleva et al. 2006; Wang and Cooper 2007; Li et al. 2016; Scotti and Swanson 2016; Verma et al. 2018; Taylor and Lee 2019). For example, mutations affecting snRNP-associated proteins PRPF3, PRPF4, PRPF6, PRPF8, PRPF31, and SNRNP200 have been identified in patients with autosomal dominant retinitis pigmentosa, a condition that results in retinal degeneration and eventual blindness (Scotti and Swanson 2016; Ruzickova and Stanek 2017). Mutations in *RNU12* (encoding the U12 snRNA) that lead to reduced expression of U12 snRNA are linked to early-onset cerebellar ataxia (Elsaid et al. 2017) and amyotrophic lateral sclerosis (Ishihara et al. 2013). Mutations in *RNU4ATAC* (encoding the U4atac snRNA) are linked to cases of Roifman syndrome (Merico et al., 2015) and microcephalic osteodysplastic primordial dwarfism type I (Edery et al. 2011; He et al. 2011; Scotti and Swanson 2016; Verma et al. 2018). These mutations affect the formation of the U4atac/U6atac.U5 tri-snRNP (Verma et al. 2018). However, patient cells exhibit some correctly spliced mRNAs indicating that these mutations cause only a partial loss of U12 spliceosome function.

**Table 1:** Human diseases associated with mutations in spliceosome genes

Gene	OMIM prevalent mutation sites	Spliceosome complex	Disease	Inheritance	References
*SNRPB*	Various mutations in an alternatively spliced regulatory exon	Sm	Cerebro-costo-mandibular syndrome	AD	(Lynch et al. 2014; Bacrot et al. 2015; Tooley et al. 2016)
*SNRPE*	c.1A>G (p.M1?); c.133G>A (p.G45S)	Sm	Hypotrichosis 11	AD	(Pasternack et al. 2013)
*SF3B1*	Mutations clustered in exons 12 to 15; p.K700E; Other hotspots: p.E622; p.R625; p.H662; p.K666; p.I704; G742	U2 snRNP	MDS; CMML; AML; solid tumors		(Papaemmanuil et al. 2011; Quesada et al. 2011; Wang et al. 2011; Harbour et al. 2013)
*SF3B4*	None prevalent; various *de novo* mutations occur at several sites	U2 snRNP	Nager acrofacial dysostosis	AD	(Bernier et al. 2012; Petit et al. 2014)
*EFTUD2*	Several either *de novo* 17q21.31 deletions encompassing *EFTUD2* or *de novo* heterozygous *EFTUD2* mutations	U5 snRNP	Mandibulofacial dysostosis type Guion-Almeida	AD	(Wieczorek et al. 2009; Lines et al. 2012)
*PRPF6*	c.2185C>T (p.R729W)	U5 snRNP	RP	AD	(Tanackovic et al. 2011a)
*PRPF8*	Various sites clustered within exon 42	U5 snRNP	RP; MDS	AD	(McKie et al. 2001; Towns et al. 2010; Maubaret et al. 2011; Kurtovic-Kozaric et al. 2015; Ruzickova and Stanek 2017)
*SNRNP200*	c.2653C>G (p.Q885E)	U5 snRNP	RP	AD	(Zhao et al. 2006; Liu et al. 2012)
*TXNL4A*	Most patients are compound heterozygous for a 34-bp deletion in the *TXNL4A* promoter (chr18:77,748,581-77,748,614del, GRCh37) and a truncating point mutation (e.g., c.131delT [p. Val44AlafsTer48] or another deletion (e.g., Ex3DEL)	U5 snRNP	Burn-McKeown syndrome	AR	(Wieczorek et al. 2014)
*PRPF3*	Various (e.g., c.1482C>T [p.T494M]; c.1478C>T [p.P493S])	U4/U6 snRNP	RP	AD	(Chakarova et al. 2002; Tanackovic et al. 2011b)
*PRPF4*	c.944C>T (p.P315L); c.-114_-97del	U4/U6 snRNP	RP	AD	(Chen et al. 2014)
*PRPF31*	Various	U4/U6 snRNP	RP	AD	(Vithana et al. 2001; Deery et al. 2002; Rivolta et al. 2006)
*RNPC3*	Compound heterozygosity (p.P474T and p.R502*)	U11/U12 snRNP	Isolated growth hormone deficiency, type V	AR	(Argente et al., 2014)
*RNU4ATAC*	Various	U4atac snRNP	Lowry-Wood syndrome; Roifman syndrome; Microcephalic osteodysplastic primordial dwarfism type I	AR	(Edery et al., 2011; He et al., 2011; Merico et al., 2015; Scotti and Swanson, 2016)
*PUF60*	Various	U2 snRNP; A complex	Verheij syndrome	AD	(Dauber et al., 2013; Low et al., 2017)
*U2AF1*	c.101G>A (p.S34F); c.101G>T (p.S34Y); c.470T>C (p.Q157R)	U2 snRNP; A complex	MDS		(Graubert et al., 2011)
*RBM10*	Various	Recruited at A complex	TARP syndrome	XLR	(Johnston et al., 2010)
*PQBP1*	Various	PRP19 complex; B complex	Renpenning syndrome	XLR	(Kalscheuer et al., 2003)
*CWC27*	Various	Recruited at B^act^ complex	RP with or without skeletal anomalies	AR	(Xu et al., 2017)
*DHX16*	Various	Recruited at B^act^ complex	Neuromuscular disease and ocular or auditory anomalies with or without seizures	AD	(Paine et al., 2019)
*PRCC*	Reciprocal translocation between chromosomes X and 1, t(X;1)(p11.2;q21.2) creating a *PRCC-TFE3* hybrid transcript	Recruited at B^act^ complex	Papillary renal cell carcinoma		(Sidhar et al., 1996)
*RNF113A*	Various	Recruited at B^act^ complex	Nonphotosensitive trichothiodystrophy 5	XLD	(Corbett et al., 2015)
*CXORF56*	c.159_160insTA (p.D54*)	Recruited at C complex	Mental retardation, X-linked 107	XL	(Verkerk et al., 2018)
*DDX41*	Various	Recruited at C complex	Susceptibility to familial myeloproliferative/lymphoproliferative neoplasms (e.g., MDS and AML)	AD	(Polprasert et al., 2015)
*EIF4A3*	Expanded 16-repeat allele (initial CACA-20-nt motif followed by 13 repeats of CGCA-20-nt, 1 CACA-20-nt, and 1 final CA-18-nt motif) in the 5′ UTR	EJC/mRNP	Robin sequence with cleft mandible and limb anomalies	AR	(Favaro et al., 2014)
*RBM8A*	200-kb deletion on 1q21.1 plus an additional *RBM8A* mutation (site varies; e.g., c.-21G>A)	EJC/mRNP	Thrombocytopenia-absent radius syndrome	AR	(Albers et al., 2012)
*FUS*	Various	hnRNP	ALS		(Reber et al., 2016)
*HNRNPA1*	c.941A>T (p.D314V); c.940G>A (p.D314N); c.956A>G (p.N319S)	hnRNP	Inclusion body myopathy with early-onset Paget disease without frontotemporal dementia 3; ALS	AD	(Kim et al., 2013)
*SMN1*	Various	All Sm snRNPs	Spinal muscular atrophy; myeloid neoplasms	AR	(Li et al., 2014; Yoshida and Ogawa, 2014; Malcovati et al., 2015; Verma et al., 2018)

*AD* autosomal dominant, *ALS* amyotrophic lateral sclerosis, *AML* acute myeloid leukemia, *AR* autosomal recessive, *CMML*, chronic myelomonocytic leukemia, *EJC* exon junction complex, *hnRNP* heterogeneous nuclear ribonucleoprotein, *MDS* myelodysplastic syndromes, *mRNP* messenger ribonucleoprotein, *OMIM* Online Mendelian Inheritance in Man^®^, *RES* retention and splicing, *RP* retinitis pigmentosa, *XLD* X-linked dominant, *XLR* X-linked recessive

## Aberrant splicing in cancer

Aberrant splicing is observed in many types of cancers, including pancreatic (Bailey et al. 2016), lung (Imielinski et al. 2012; Brooks et al. 2014), breast (Maguire et al. 2015; Nik-Zainal et al. 2016), colorectal (Adler et al. 2014), uveal melanoma (Furney et al. 2013; Harbour et al. 2013; Martin et al. 2013), acute myeloid leukemia (AML), chronic myelomonocytic leukemia (CMML), and myelodysplastic syndromes (MDS) (Graubert et al. 2011; Malcovati et al. 2011; Papaemmanuil et al. 2011, 2016; Quesada et al. 2011; Rossi et al. 2011; Wang et al. 2011; Yoshida et al. 2011; Bejar et al. 2012; Damm et al. 2012; Visconte et al. 2012a, 2012b; Scott and Rebel 2013; Ilagan et al. 2015; Kim et al. 2015; Lindsley et al. 2015; Madan et al. 2015; Shirai et al. 2015; Scotti and Swanson 2016; Desai et al. 2018). Splicing alterations in cancer cells often contribute to cancer progression (Rahman et al. 2020).

**Figure 4:**
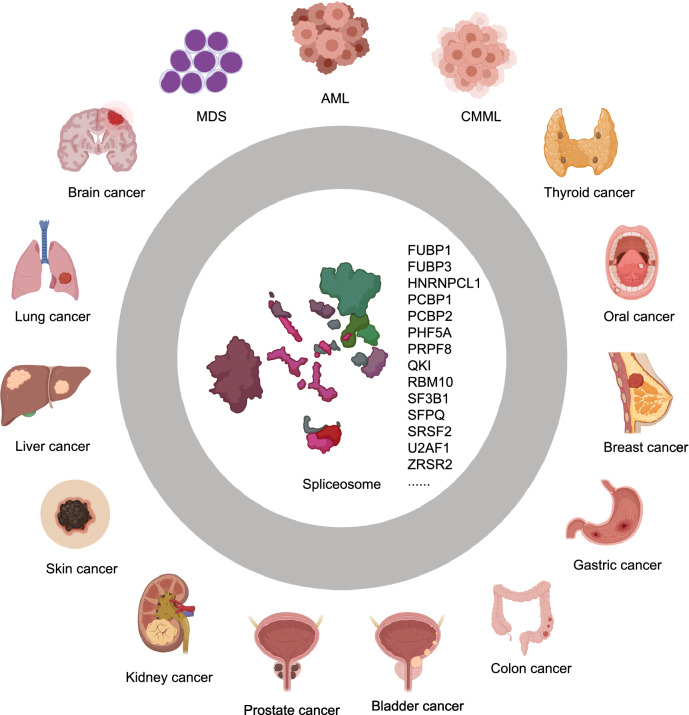
Schematic representation of human cancers linked to mutations or abnormal expressions of splicing factors and spliceosome genes. Mutated or aberrantly expressed splicing factors and spliceosome genes including *FUBP1*, *FUBP3*, *HNRNPCL1*, *PCBP1*, *PCBP2*, *PHF5A*, *QKI*, *RBM10*, *SF3B1*, and *SFPQ*, are linked to a variety of solid tumors. While alterations in some other spliceosome genes, e.g., *PRPF8*, *SF3B1*, *SRSF2*, *U2AF1*, and *ZRSR2*, are associated with hematologic disorders and malignancies, such as myelodysplastic syndromes (MDS), acute myeloid leukemia (AML), and chronic myelomonocytic leukemia (CMML)

Splicing alterations can result from several mechanisms, including differential regulation during transcription, alternative splicing, nonsense-mediated decay, microRNAs (miRNAs), long non-coding RNAs (lncRNAs), and aberrant post-translational modifications due to cancer-related changes in intracellular signaling (Urbanski et al. 2018). Splicing alterations can also be due to mutations in spliceosome components or splicing factors. Any of the above alterations can subsequently result in altered function and/or nuclear-cytoplasmic localization of the splicing factors, splicing changes to downstream targets of the splicing factors, changes in cellular signaling in tumor cells, and changes in tumor initiation and progression (Fig. 4) (Zhang and Manley 2013; Dvinge et al. 2016).

### Transcription

Transcription of splicing factors and spliceosome proteins can be differentially regulated by several factors in cancer cells. For example, the transcription factor and oncogene MYC regulates the transcription of splicing factors (e.g., HNRNPA1, PTBP1) (Zhang et al. 2016a) and the core snRNP particle assembly genes (e.g., PRMT5) in cancer cells (Koh et al. 2015), consequently leading to enhanced cell survival. The Wnt signaling pathway also controls the transcriptional activation of splicing factors in some cancers (Goncalves et al. 2008; Corbo et al. 2012).

### Alternative splicing

RNA-binding protein expression can be regulated through the splicing of their pre-mRNAs. Members of the SR protein family (e.g., SRSF1, SRSF3, etc.) are able to regulate the inclusion of premature stop codon-containing exons within their own mRNAs. These aberrant transcripts subsequently undergo nonsense-mediated decay, reducing the expression levels of SR protein (Jumaa and Nielsen 1997; Sureau et al. 2001). The splicing of some RNA-binding factors can also be regulated by other splicing factors such as RBFOX2 in cancer tissues, resulting in altered function or transcript degradation (Jumaa and Nielsen 1997; Rossbach et al. 2009; Venables et al. 2013).

### MicroRNAs

MiRNAs are small single-stranded noncoding RNAs that can bind target mRNAs, promoting the target mRNA’s degradation. Splicing factors are among the many targets of miRNAs. SRSF7 expression is suppressed by the miRNAs miR-30a-5p and miR-181a-5p in renal tumors, subsequently leading to altered splicing of apoptosis regulators, oncogenes, and tumor suppressors (Boguslawska et al. 2016). In tumors, the expression of splicing factor SRSF1 is putatively increased due to lymphoma/leukemia-related factor-associated repression of miR-28 and miR-505 (Verduci et al. 2010). Also, retinoic acid-induced miR-10a and miR-10b upregulation in neuroblastoma cells, and subsequent repression of SRSF1, putatively results in terminal differentiation of neuroblastoma cells (Meseguer et al. 2011).

### Long non-coding RNAs

LncRNAs facilitate splicing factors’ binding to exonic or intronic splicing silencer elements to regulate alternative splicing (Urbanski et al. 2018). The lncRNAs *PCGEM1* and *BC200* interact with splicing factors HNRNPA1, U2AF65 or HNRNPA2B1 to regulate the alternative splicing of *AR* (Zhang et al. 2016b) and *BCL-x* (Singh et al. 2016)*.* Some lncRNAs (e.g., *MALAT1*) also regulate alternative splicing by regulating nuclear localization of SR proteins (Tripathi et al. 2010). The lncRNA *LINC01133* promotes nuclear sequestration of SRSF6, preventing SRSF6-associated epithelial-mesenchymal transition and metastasis in colorectal cancer mouse models (Kong et al. 2016).

### Post-translational modifications

Post-translational modifications affect the localization and function of many proteins, including splicing factors. For example, SR protein phosphorylation by SR-specific protein kinases (SRPKs) regulates SR activation and SR-mediated splicing. Aberrant SR protein phosphorylation (either hypo- or hyper-phosphorylation) inhibits splicing (Zhong et al. 2009). SRPK localization is also regulated by its phosphorylation, which promotes nuclear import through interaction with the nuclear import receptor transportin-SR2 (Koizumi et al. 1999; Lai et al. 2000). The CDC-like kinase CLK2 alters splicing, putatively by altering the function of SR proteins through phosphorylation (Yoshida et al. 2015). AKT phosphorylates SRSF1, SRSF7, and SRSF5 as well as SRPKs (Blaustein et al. 2005). The AKT-SRPK-SR axis promotes epidermal growth factor signaling and regulation of alternative splicing (Zhou et al. 2012).

### Mutations in spliceosome components and splicing factors

Splicing alterations in cancers can also be caused by mutations affecting splicing-regulatory elements or splicing factors. The most common spliceosome-associated mutations in cancers are in *SF3B1*, *SRSF2*, *U2AF1*, and *ZRSR2*. Mutations in these genes result in changes in the RNA recognition preferences of the encoded proteins (Rahman et al. 2020); more information about these proteins in cancer can be found in the next section. Recurrent mutations in cancer have been identified in several RNA splicing factors (e.g., *PRPF8*, *RBM10*, *SFPQ*, *PHF5A*, *HNRNPCL1*, *PCBP1*, *PCBP2*, *FUBP1*, *FUBP3*, and *QKI)*, but these mutations have not been fully functionally characterized (Rahman et al. 2020).

Recent studies have examined aberrant spliceosome component function in different types of cancers. Mutations in the U1 snRNA have been reported in medulloblastoma, chronic lymphocytic leukemia, hepatocellular carcinoma, B cell non-Hodgkin lymphoma, and pancreatic adenocarcinoma (Suzuki et al. 2019). Mutations at the fifth nucleotide (r.5A>G) of the U11 snRNA gene (*RNU11*) have been identified in medulloblastoma (Suzuki et al. 2019). Tumors expressing mutant snRNAs exhibit aberrant splicing, notably excess cryptic 5′ splice site events (Rahman et al. 2020).

Compared to hematological cancers that often exhibit point mutations in core spliceosome genes (Lee and Abdel-Wahab 2016), few recurrent mutations in spliceosome genes have been detected in prostate cancer (Sebestyen et al. 2016). Instead, splicing changes, namely intron retention, in prostate cancer cells are attributed to copy number variation of splicing-regulatory genes (Zhang et al. 2020). Prostate cancer cells exhibit variations in the genomic copy number of almost 70% of genes encoding spliceosome core subunits and auxiliary splicing regulatory proteins during prostate cancer development and progression (Zhang et al. 2020). These splicing alterations correlate with disease progression, prostate cancer stemness, and tumor aggressiveness (Zhang et al. 2020).

The spliceosome component RNF113A is overexpressed in pulmonary adenocarcinomas (Shostak et al. 2020). RNF113A promotes cell survival in lung cancer cells treated with the chemotherapeutic drug Cisplatin by promoting the splicing of prosurvival candidate genes *SAT1* and *NUPR1*. RNF113A also stabilizes the prosurvival protein MCL1 by an unknown mechanism, putatively through a spliceosome-independent manner. Loss of RNF113A expression presumably enhances cell death through MCL1 downregulation.

## Spliceosome Mutations are frequently observed in myelodysplastic syndromes

MDS are a group of serious cancers in which the bone marrow can not produce enough healthy and mature blood cells (Heaney and Golde 1999; Pellagatti and Boultwood 2015). Early on, there are typically no symptoms. As the disease progresses, symptoms may include feeling tired, shortness of breath, easy bleeding, or frequent infections. Patients with MDS show increased numbers of bone marrow myeloblasts (i.e., immature cells) over time and are at risk of developing AML (Heaney and Golde 1999; Pellagatti and Boultwood 2015; Bersanelli et al. 2021).

Approximately 50% of MDS patients have a mutation in *SF3B1*, *U2AF1*, *SRSF2*, or *ZRSR2* (Yoshida et al. 2011; Haferlach et al. 2014; Nguyen et al. 2018). Mutations in *SRSF2* and *U2AF1* are the most frequent splicing-associated mutations found in the more aggressive subtypes of MDS, including refractory anemia with excess blasts I (RAEB I) and RAEB II (Inoue et al. 2016). The MDS-associated spliceosome mutations identified thus far are heterozygous mutations as opposed to nonsense mutations, indicating that these mutations are neomorphic or dominant-negative (Inoue et al. 2016).

*SF3B1* encodes a protein that stabilizes U2 snRNP binding to the branch point sequence (Yip et al. 2016). Greater than 90% of refractory anemia with ringed sideroblasts (RARS) MDS patients and approximately 70% of non-RARS forms of MDS with ringed sideroblasts (i.e., refractory cytopenia with multilineage dysplasias; ringed sideroblasts and RARS associated with marked thrombocytosis) have mutations in *SF3B1* (Inoue et al. 2016). MDS-causing *SF3B1* mutations result in aberrant 3′ splice site usage, leading to coding of premature stop codons and subsequent nonsense-mediated decay of the affected transcripts (Darman et al. 2015).

*U2AF1*-encoded protein recognizes the AG dinucleotide at 3′ splice sites (Wu et al. 1999; Yip et al. 2016). Mutations in *U2AF1* have been identified not only in patients with MDS (Graubert et al. 2011; Yoshida et al. 2011), but also in patients with CMML, secondary acute AML (Graubert et al. 2011; Yoshida et al. 2011; Papaemmanuil et al. 2013), hairy cell leukemia (Waterfall et al. 2014), pancreatic ductal adenocarcinomas (Bailey et al. 2016), and non-small cell lung adenocarcinomas (Imielinski et al. 2012). The cancer-causing mutations in *U2AF1* typically affect either S34 or Q157 within the zinc finger domains of the U2AF1 protein, resulting in changes in U2AF1 3′ splice site recognition. Furthermore, a U2AF1 mutation (p.S34F) causes accumulation of a transcription intermediate containing RNA:DNA hybrids and single-stranded DNA (i.e., R loops) (Nguyen et al. 2018). The R loops elicit a DNA damage response induced by ataxia telangiectasia mutated- and Rad3-related kinase (ATR). S34F mutant U2AF1-expressing cells undergo ATR inhibitor-induced cell death, which also promotes DNA damage. The U2AF1 mutations cause mis-splicing of hundreds of transcripts by exon skipping, exon inclusion, or alternative 3′ splice site usage (Ilagan et al. 2015; Okeyo-Owuor et al. 2015; Shirai et al. 2015).

*SRSF2* encodes a splicing factor that binds mRNA exon splicing enhancer motifs (Yip et al. 2016). *SRSF2* mutations are linked to poor prognosis and a higher risk of transformation in AML (Damm et al. 2012; Meggendorfer et al. 2012; Zhang et al. 2012; Papaemmanuil et al. 2013). *SRSF2* mutations that affect P95 near the RNA recognition motif in the SRSF2 protein result in changes to SRSF2’s RNA-binding characteristics, altered splicing patterns, and impaired hematopoietic cell differentiation (Kim et al. 2015; Komeno et al. 2015; Zhang et al. 2015).

*ZRSR2* encodes a spliceosome component required for the recognition of the 3′ splice site for both U2- and U12-type introns. *ZRSR2* is an X-linked gene; therefore, mutations in *ZRSR2* are predominantly linked to MDS in male patients (Yoshida et al. 2011). The *ZRSR2* mutations are sporadic across the entire coding region. These loss-of-function mutations lead to increased retention of U12 introns, but not U2 intron splicing. Notably, loss-of-function mutations in *ZRSR2* cause widespread minor intron retention and thus enhance hematopoietic stem cell self-renewal as well as drive diverse cancer predisposition, which putatively results from *LZTR1* minor intron retention (Inoue et al. 2021).

## Anti-cancer strategies targeting the spliceosome machinery

Mutations affecting the spliceosome or splicing factors result in perturbation in downstream splicing targets, many of which are in signaling pathways involved with cancer. Alternatively, the copy number or expression level of a splicing factor can be changed in tumors without the presence of a mutation (Anczukow and Krainer 2016). Therefore, identifying anti-cancer strategies towards problematic factors is imperative.

Antisense oligonucleotides (ASOs) are synthetic, single-stranded oligodeoxynucleotides that inhibit gene expression by binding cellular RNA. ASOs can also be developed to target non-coding RNAs, including lncRNAs and miRNAs. ASOs can be used to alter splicing, namely the selective removal or inclusion of a particular exon (Spitali and Aartsma-Rus 2012; Havens and Hastings 2016). These ASOs alter splicing by changing the spliceosome recognition sites on the target RNAs. ASOs can potentially restore splice defects in mutated genes, allowing a normal protein to be expressed (Dominski and Kole 1993). Numerous ASOs (e.g., Danvatirsen, Trabederen, Custirsen) designed for different oncogenic targets (e.g., STAT3, TGFB2, clusterin, respectively) are being investigated in clinical trials or are in development (Quemener et al. 2020).

Small molecules that target components of the spliceosome have exhibited antitumor effects in cancer cells with spliceosome component mutations (Bonnal et al. 2020). For example, prostate cancer cells and the prostate cancer cell line PC3 show sensitivity to the spliceosome inhibitor E7107 (Zhang et al. 2020). E7107 targets SF3B complex, preventing tight binding of the U2 snRNP to pre-mRNA (Kotake et al. 2007; Folco et al. 2011). Treatment with E7107 suppresses prostate cancer cell migration and invasion as well as causes cell death by promoting cell cycle arrest at the G_2_/M phase. E7107 putatively inhibits the splicing of pancreatic cancer-promoting genes, consequently resulting in a less aggressive phenotype. Yet more preclinical studies are necessary to determine which cancer patients would most benefit from these therapies, as well as to determine the combination strategies with other therapies (Eymin 2020).

Although small-molecule splicing modulators have been explored as anti-cancer therapeutics for a lone time (Kaida et al. 2007; Kotake et al. 2007), the mechanisms underlying the selective antitumor activity remain largely unknown. Recent studies have shown that spliceosome-targeted therapies (STTs) can cause widespread cytoplasmic accumulation of mis-spliced (e.g., intron-retained) mRNAs, many of which form double-stranded RNAs (dsRNAs). Consequently, these dsRNAs can be recognized by intracellular immune sensors, initiating antiviral signaling (viral mimicry) and extrinsic apoptosis in breast cancer (Bowling et al. 2021; Ishak et al. 2021).

## Cancer-associated spliceosome mutations result in aberrant immune signaling

MDS patients often exhibit inflammation due to the overproduction of inflammatory cytokines by myeloid cells. Subsequently, the excess cytokines could contribute to impaired hematopoietic stem cell niche function and the suppression of normal hematopoiesis (Baldridge et al. 2011). Altered splicing of inflammatory and immune genes (e.g., *IRAK4*, *MAP3K7*, and *CASP8*) due to spliceosome mutations may contribute to MDS pathogenesis by leading to inflammation, changes in immune cell function, and increased risk of infection (Starczynowski and Karsan 2010b, 2010a; Pagano and Caira 2012; Darman et al. 2015; Ilagan et al. 2015; Kim et al. 2015; Zhang et al. 2015; Alsafadi et al. 2016; Grignano et al. 2018; Pollyea et al. 2019; Smith et al. 2019). For example, in some MDS/AML patients with *U2AF1* mutations, *IRAK4* is alternatively spliced to retain exon 4. This alternatively spliced *IRAK4* subsequently encodes a protein (termed IRAK4-L) that assembles with the myddosome, resulting in maximal activation of NF-κB. This process is essential for leukemic cell function, and inhibition of IRAK4-L blocks leukemic growth.

Inhibition of *SF3B1*, *SF3A1*, *SF3A2*, *SF3A3*, *U2AF1*, *SRSF2*, or *EFTUD2* in mouse or human macrophages results in diminished toll-like receptor (TLR)-induced inflammatory cytokine production after stimulation with the TLR4 agonist lipopolysaccharide (De Arras and Alper 2013; De Arras et al. 2014; O'Connor et al. 2015; Lv et al. 2019; Pollyea et al. 2019). Notably, knockdown of any of the genes does not affect cell viability or phagocytosis (De Arras and Alper 2013; De Arras et al. 2014; O'Connor et al. 2015). Conversely, expression of *SF3B1*, *U2AF1*, and *SRSF2* MDS-associated mutations in macrophages, MDS patient-derived cell lines, and mouse and human myeloid cells results in enhanced NF-ĸB activation and subsequent lipopolysaccharide-induced inflammatory cytokine production (Pollyea et al. 2019). These inflammatory changes point to a mechanism that contributes to MDS pathogenesis.

MyD88 is an adaptor protein that functions in most TLR signaling pathways. Knockdown of *Sf3a1*, *Sf3b1*, *Srsf2* or *U2af1* in RAW264.7 mouse macrophage cells results in increased expression of the anti-inflammatory short form of MyD88 (MyD88s) and subsequently reduced inflammatory cytokine production; the expression level of the long isoform of MyD88 (MyD88L) is unchanged (O'Connor et al. 2015; Pollyea et al. 2019). Interestingly, *MYD88* splicing is not affected in the myeloid leukemia cell line K562 upon splicing factor knockdown, indicating that the splicing of other TLR signaling genes is promoting the increased inflammatory cytokine production in this cell type.

TAK1 (alternatively, MAP3K7) functions as a regulator of NF-ĸB, JNK, and MAPK signaling pathways. A mutation in *SF3B1* (p.K700E) can cause aberrant splicing of *MAP3K7* (encoding TAK1) (Lee et al. 2018). Expression of mutant *SF3B1* in K562 cells leads to increased production of the alternative TAK1 isoform and enhanced innate immune signaling; however, the expression of canonical TAK1 is unchanged (Lee et al. 2018; Pollyea et al. 2019).

Conditional deletion of *Srsf1* results in severe defects at late stage thymocyte development, thus a notable reduction in periphery T cell pool. SRSF1 can directly bind and regulate *Irf7* and *Il27ra* expression via alternative splicing in response to type I interferon signaling (Qi et al. 2021). SRSF1 restrains IFN-γ production and Th1 differentiation through the control of RhoH (Katsuyama et al. 2021). SRSF1 modulates the expression of several genes involved in immune system functions through multiple mechanisms (Paz et al. 2021).

Moreover, cells expressing a mutant *SRSF2* (p.P95H) can express an alternative caspase 8 isoform that is truncated due to exon 6 skipping. Expression of the alternative caspase 8 isoform results in enhanced NF-ĸB activity (Lee et al. 2018; Pollyea et al. 2019).

## The expanding role of core spliceosome components in immunity

**Figure 5:**
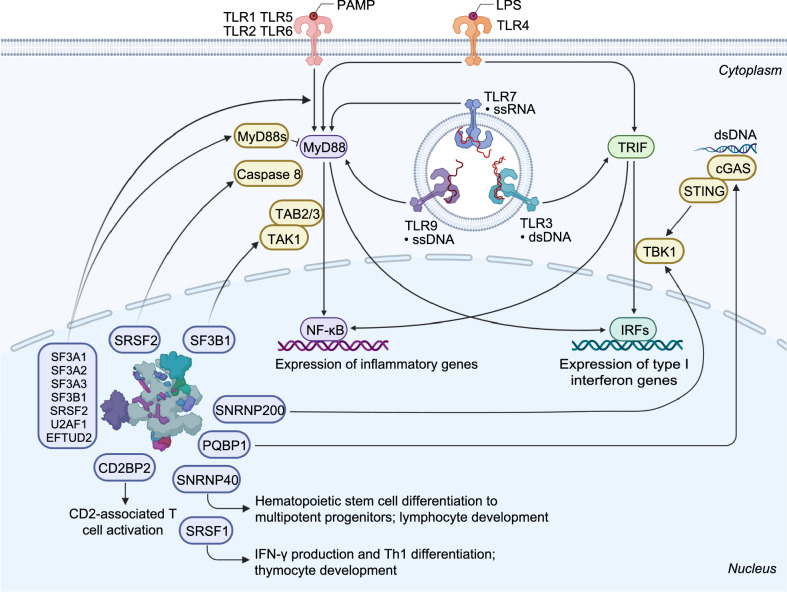
Schematic representation of immune functions linked to splicing factors and spliceosome components. The spliceosome proteins (e.g., SF3A1, SF3A2, SF3A3, SF3B1, SRSF2, U2AF1, and EFTUD2) can regulate TLR-induced NF-ĸB activation and inflammatory cytokine production. Knockdown of *Sf3a1*, *Sf3b1*, *Srsf2*, or *U2af1* in mouse macrophage cells results in increased expression of the anti-inflammatory short form of MyD88 (MyD88s) and subsequently reduced inflammatory cytokine production. SF3B1 can also regulate the production of an alternative TAK1 isoform, subsequently affecting innate immune signaling. SRSF1 restrains IFN-γ production and Th1 differentiation; it regulates the late stage of thymocyte development and the expression of several immune-related genes through multiple mechanisms. SRSF2 regulates the expression of an alternative caspase 8 isoform and subsequent NF-ĸB activity. SNRNP40 plays a pivotal role in hematopoietic stem cell differentiation to multipotent progenitors and further to common lymphoid progenitors. SNRNP200 activates IRF3-mediated antiviral innate immune responses through an interaction with TBK1. CD2BP2 is involved in CD2-associated T cell activation via binding to the cytoplasmic tail of CD2 molecule. PQBP1 can interact with cGAS to trigger type I interferon production in response to cytosolic nucleic acids or DNA damage

Several snRNP complex components are differentially expressed in a cell- or tissue-specific manner, and this often correlates with tissue-specific phenotypes that arise upon their inactivation. In addition, core spliceosome components such as SNRNP40, SNRNP200, CD2BP2, and PQBP1, harbor splicing-independent functions specifically related to immune signaling (Fig. 5).

### SNRNP40

Our recent studies have shown that a viable hypomorphic mutation in mouse spliceosome gene *Snrnp40* causes remarkable immune phenotypes (Zhang et al. 2019). SNRNP40 is part of the U5 snRNP; however, its exact function is unknown. In the adult mouse, the SNRNP40 protein is predominantly expressed in lymphoid tissue. The *Snrnp40* mutant mice show impaired hematopoietic stem cell differentiation to multipotent progenitors, and aberrant multipotent progenitor differentiation to common lymphoid progenitors, T cells, B cells and natural killer cells. A few hundred splicing errors, mostly intron retention, can be observed in the *Snrnp40*-deficient mouse hematopoietic stem and progenitor cells or T cells. These cumulative splicing errors lead to reduced expression of immune-related proteins and subsequent immune phenotypes (Zhang et al. 2019). Furthermore, the SNRNP40 protein also shows localization within the cytoplasm (unpublished data), implying its splicing-independent functions, most likely in immune signaling due to the severe immune-specific phenotypes observed in the *Snrnp40* mutant mice.

### SNRNP200

TBK1 is a downstream kinase activated by a number of pattern recognition receptor (PRR)–adaptor protein pairs, including cGAS-STING, RIG-I–MAVS, and TLR3/4-TRIF. TBK1 functions as an adaptor to subsequently promote the expression of proinflammatory and antiviral cytokines and chemokines (Liu et al. 2015). The spliceosome protein SNRNP200 which catalyzes the ATP-dependent unwinding of U4/U6 RNA duplices is able to bind RNA and interact with TBK1. Upon viral infection, SNRNP200 relocalizes into TBK1-containing cytoplasmic structures, and subsequently promotes the activation of IRF3-mediated antiviral innate immune response. Loss of SNRNP200 expression results in a decrease in antiviral innate immunity (Tremblay et al. 2016).

### CD2BP2

The adaptor protein CD2BP2 is a constituent of the U5 snRNP (Kofler et al. 2004; Laggerbauer et al. 2005). Yeast two-hybrid analysis determines that CD2BP2 binds the cytoplasmic tail of CD2, a surface antigen that promotes the adhesion and activation of T cells upon engagement with its ligand (Nishizawa et al. 1998; Heinze et al. 2007). The CD2BP2 and CD2 interaction regulates CD2-associated T cell activation (Freund et al. 2002; Heinze et al. 2007), and transient transfection of a CD2BP2 fragment in Jurkat cells induces CD2-stimulated IL-2 production (Nishizawa et al. 1998). Conversely, knockdown of CD2BP2 expression does not impact cytokine expression in primary T cells in response to CD2 stimulation, indicating that CD2 signaling is not completely dependent on CD2BP2 binding (Heinze et al. 2007).

### PQBP1

Polyglutamine binding protein 1 (PQBP1) is a component of the B complex in spliceosome. PQBP1 can also associate with, and inhibit, the DNA sensors cyclic GMP-AMP synthase (cGAS) and interferon-γ-inducible protein 16 (IFI16) in response to cytosolic DNA. Reduced expression of PQBP1 in THP-1 cells leads to increased type I interferon production in response to transfected cytosolic nucleic acids or DNA damage (Shannon et al. 2018). Furthermore, PQBP1 directly binds to reverse-transcribed HIV-1 DNA and interacts with cGAS to trigger an IRF3-dependent innate immune response. Primary human monocyte-derived DCs from Renpenning syndrome patients who harbor *PQBP1* mutations exhibit a significantly reduced innate response to HIV-1 challenge, emphasizing the role of PQBP1 as a proximal innate sensor of HIV-1 infection (Yoh et al. 2015).

## Perspectives

The immune-related findings of SNRNP200 and PQBP1 highlight that some spliceosome components can have tissue-specific functions within the cytoplasm (Tremblay et al. 2016; Shannon et al. 2018). The observation that the SNRNP40 protein also localizes outside of the nucleus suggests that additional spliceosome-independent functions of SNRNP40 are worth studying. Other spliceosome proteins with documented splicing-independent functions include survival of motor neuron 1 (SMN1) and splicing factor 3b subunit 4 (SF3B4). In the spliceosome, SMN1 functions in the early stages of cytoplasmic snRNP assembly by promoting the addition of the Sm proteins around the snRNA (Tisdale et al. 2013; Li et al. 2014). SMN1 also functions in the trafficking of mature mRNA within the cytoplasm of axons and neurites (Fallini et al. 2016). SF3B4, a subunit of the U2 snRNP, putatively functions in transcription (Devotta et al. 2016; Marques et al. 2016), translation (Ueno et al. 2019), and cell signaling (Nishanian and Waldman 2004). In *Arabidopsis*, SF3B4 mediates the transcription of certain genes by recruiting the RNA polymerase II complex to the RNA transcript (Xiong et al. 2019); however, the role of SF3B4 in human transcription regulation remains unclear. While during translation, SF3B4 interacts with the cofactor p180, promoting the association of mRNAs with the endoplasmic reticulum membrane and assembly of polyribosomes (Ueno et al. 2019). SF3B4 regulates the receptor kinase BMPR1A-associated signaling and specifically inhibits osteochondral cell differentiation. SF3B4 interacts with, and downregulates expression of BMPR1A at the cell surface (possibly by regulating receptor internalization), subsequently regulating craniofacial development in embryos (Nishanian and Waldman 2004; Watanabe et al. 2007).

Future studies will undoubtedly show that other spliceosome components display splicing-independent functions within the nucleus and the cytoplasm, including the functions that may be related to innate immunity, cell cycle, cell growth, cell death, or transformation. Moreover, future studies will uncover new correlations between spliceosome mutations, aberrant splicing, immune dysregulation, and human cancers.

## Abbreviations

AML: acute myeloid leukemia
AR: androgen receptor
ASOs: antisense oligonucleotides
ATR: ataxia telangiectasia-mutated and RAD3-related kinase
BMPR1A: bone morphogenetic protein receptor type 1A
CD2BP2: CD2 cytoplasmic tail binding protein 2
cGAS: cyclic GMP-AMP synthase
CLK2: CDC-like kinase 2
CMML: chronic myelomonocytic leukemia
DDX23: DEAD-box helicase 23
DDX46: DEAD-box helicase 46
DHX8: DEAH-box helicase 8
DHX15: DEAH-box helicase 15
DHX16: DEAH-box helicase 16
DHX38: DEAH-box helicase 38
EFTUD2: elongation factor Tu GTP binding domain containing 2
FUBP1: far upstream element binding protein 1
FUBP3: far upstream element binding protein 3
HIV-1: human immunodeficiency virus 1
HNRNP: heterogeneous nuclear ribonucleoprotein
IFI16: interferon-γ-inducible protein 16
IRAK4: interleukin 1 receptor associated kinase 4
JNK: JUN N-terminal kinase
LZTR1: leucine zipper like transcription regulator 1
MAPK: mitogen-activated protein kinase
MAP3K7: mitogen-activated protein kinase kinase kinase 7
MAVS: mitochondrial antiviral signaling protein
MCL1: MCL1 apoptosis regulator, BCL2 family member
MDA5/IFIH1: interferon induced with helicase C domain 1
MDS: myelodysplastic syndromes
MyD88: myeloid differentiation primary response 88
NF-κB: nuclear factor kappa B
NUPR1: nuclear protein 1, transcriptional regulator
PCBP1: poly(rC) binding protein 1
PCBP2: poly(rC) binding protein 2
PHF5A: PHD finger protein 5A
PQBP1: polyglutamine binding protein 1
PRMT5: protein arginine methyltransferase 5
PRPF: pre-mRNA processing factor
PTBP1: polypyrimidine tract binding protein 1
QKI: quaking homolog, KH domain RNA binding
RAEB: refractory anemia with excess blasts
RARS: refractory anemia with ringed sideroblasts
RBFOX2: RNA binding fox-1 homolog 2
RBM10: RNA binding motif protein 10
RhoH: Ras homolog family member H
RIG-I/DDX58: DExD/H-box helicase 58
RNF113A: ring finger protein 113A
RP: retinitis pigmentosa
SAT1: spermidine/spermine N1-acetyltransferase 1
SF1: splicing factor 1
SF3B1: splicing factor 3b subunit 1
SF3B4: splicing factor 3b subunit 4
SFPQ: splicing factor proline and glutamine rich
SMN1: survival of motor neuron 1
snRNP: small nuclear ribonucleoprotein
SNRNP40: small nuclear ribonucleoprotein U5 subunit 40
SNRNP200: small nuclear ribonucleoprotein U5 subunit 200
SR: serine/arginine-rich
SRPK: SRSF protein kinase
SRSF1: serine/arginine-rich splicing factor 1
SRSF2: serine/arginine-rich splicing factor 2
STAT3: signal transducer and activator of transcription 3
STING: stimulator of interferon response cGAMP interactor 1
TAK1: tGF-beta activated kinase 1
TBK1: tANK-binding kinase 1
TGFB2: transforming growth factor beta 2
TLR: toll-like receptor
TRIF/TICAM1: toll-like receptor adaptor molecule 1
U snRNA: uridine-rich small nuclear RNA
U2AF1: U2 auxiliary factor 1
U2AF2: U2 auxiliary factor 2
ZRSR2: zinc finger CCCH-type, RNA binding motif and serine/arginine rich 2
